# The Musical Emotion Discrimination Task: A New Measure for Assessing the Ability to Discriminate Emotions in Music

**DOI:** 10.3389/fpsyg.2019.01955

**Published:** 2019-08-27

**Authors:** Chloe MacGregor, Daniel Müllensiefen

**Affiliations:** Department of Psychology, Goldsmiths, University of London, London, United Kingdom

**Keywords:** music perception, music performance, emotion perception, emotional intelligence, musical training

## Abstract

Previous research has shown that levels of musical training and emotional engagement with music are associated with an individual’s ability to decode the intended emotional expression from a music performance. The present study aimed to assess traits and abilities that might influence emotion recognition, and to create a new test of emotion discrimination ability. The first experiment investigated musical features that influenced the difficulty of the stimulus items (length, type of melody, instrument, target-/comparison emotion) to inform the creation of a short test of emotion discrimination. The second experiment assessed the contribution of individual differences measures of emotional and musical abilities as well as psychoacoustic abilities. Finally, the third experiment established the validity of the new test against other measures currently used to assess similar abilities. Performance on the Musical Emotion Discrimination Task (MEDT) was significantly associated with high levels of self-reported emotional engagement with music as well as with performance on a facial emotion recognition task. Results are discussed in the context of a process model for emotion discrimination in music and psychometric properties of the MEDT are provided. The MEDT is freely available for research use.

## Introduction

The affective experience associated with music is commonly considered a key motive for engagement in musical activities (Juslin and Laukka, 2004). Music is often used in a constructive manner, to express emotion through composition and performance, or to evoke or regulate an emotional state through listening. The amount of research contributing to an understanding of emotional processes associated with music has increased considerably over the last few decades, most of which has focused especially on the expression and induction of musical emotions (Thompson, 2009). It has been suggested that the ability to perceive musical emotions may vary across individuals, just as recognition of emotional facial and vocal expressions has been found to vary according to individual differences (Palermo et al., 2013; Taruffi et al., 2017). Though many tests have been developed to detect such differences in facial and vocal recognition (Mayer et al., 2008), equivalent tests for musical emotion recognition are considerably less common. The current study therefore aims to establish a new measure of emotion discrimination using music in order to investigate whether differences in emotional, musical, and perceptual abilities may account for variation in the perception of musical emotions.

One factor of potential influence within musical emotion decoding is emotional intelligence (EI): the ability to categorize, express and regulate one’s emotions, as well as those of others (Salovey and Mayer, 1990). EI is typically separated into two constructs for the purpose of measurement; ability EI, measured using cognitive ability tests, and trait EI, assessed via self-report methods (Petrides et al., 2004). In keeping with a recent study of emotion decoding in music (Akkermans et al., 2018), a self-report measure of trait EI was used within the current research. Differences in recognition of emotion within speech prosody have been linked to EI (Trimmer and Cuddy, 2008), signifying the potential influence of EI on musical emotion decoding, especially when considering the strong evidence for a link between the communication of emotions in speech and music (Juslin and Laukka, 2003). This supposition is further endorsed by Resnicow et al. (2004), who found a positive correlation between EI and a test of emotion recognition in which participants rated basic emotions conveyed through piano pieces. Evidence therefore indicates that differences in EI may explain variation in music-perceived emotion.

One relevant component of EI of is emotional contagion (EC) (Salovey and Mayer, 1990), which refers to ones’ tendency to be influenced by, or unconsciously mimic, others’ emotional states (Doherty, 1997). EC has mostly been investigated in relation to facial expression (Juslin and Västfjäll, 2008); for example, one study examined facial muscle responses to videos of emotional singing and found that participants tended to unconsciously imitate the emotional facial expression of the singer (Chan et al., 2013). Though less prevalent in the literature, there are also reports of contagion from vocal expression (e.g., Neumann and Strack, 2000). On account of such evidence, as well as the aforementioned notion that music’s emotional quality may be derived from its similarities to vocal expression (Juslin and Laukka, 2003), it has been speculated that EC may occur in music listening through the internal mimicking of a perceived expression (Juslin et al., 2009). This is backed up by neuroimaging research conducted by Koelsch et al. (2006); when participants were exposed to music, activation was found within areas of the mirror-neuron system which have been linked with vocal production, thought to represent the mimicking of emotions expressed by music (Juslin and Västfjäll, 2008). Such evidence indicates that EC may contribute to the categorization of emotions in music.

Though a high level of emotional ability is likely to result in a consistent level of emotion processing throughout different modalities, it is arguable that emotional ability may vary specifically in relation to music. It is thus necessary to consider an individuals’ typical level of emotional engagement with music, alongside more general measures of emotional ability, when investigating factors influencing emotion recognition. Emotional music skills can be measured using the Goldsmiths Musical Sophistication Index (GOLD-MSI) (Müllensiefen et al., 2013), a self-report tool that allows for the assessment of a wide range of musical skills and behaviors. The *Emotion* subscale of the Gold-MSI subscale was used in a recent study, which found that self-reported level of emotional engagement with music predicted accuracy on a musical emotion decoding task (Akkermans et al., 2018). A high level of emotional engagement with music, as measured using the Gold-MSI, may therefore be a good indicator of ability to discriminate musical emotional expression.

Musical ability has also been explored with regard to its relationship with emotional capacity (Hallam, 2010). The idea that musical expertise may enhance emotional skills seems plausible when taking into account other cognitive advantages found to result from training (Schellenberg, 2005). Accordingly, it has been suggested that enhanced musical and acoustic processing acquired through training (Kraus and Chandrasekaran, 2010) may contribute to an enhanced sensitivity to non-verbal emotions (Taruffi et al., 2017; Akkermans et al., 2018). Empirical evidence for this claim has been provided by studies conducted by Thompson et al. (2004) and Lima and Castro (2011), both of which demonstrated that a group of musicians were better able to decode emotions in speech prosody than untrained controls. However, one study carried out by Trimmer and Cuddy (2008) found little variation among individuals’ recognition of emotions in speech prosody based on their level of musical expertise (Trimmer and Cuddy, 2008). Lima and Castro (2011) point out that Trimmer and Cuddy’s (2008) findings could be accounted for by distinctions between the participants recruited for each study. The participants in both theirs and Thompson et al. (2004) study had, on average, between 8 and 14 years of musical training, whereas participants in Trimmer and Cuddy’s (2008) study had an average of 6.5 years. As it is possible that the effects of training may only manifest at a measurable level as a result of extensive training, Lima and Castro (2011) argue that this could have played a role in the lack of a discernable effect. More recent studies investigating musical emotion decoding have uncovered a positive association between decoding performance and self-reported musical expertise, providing additional support for the influence of musical training (Taruffi et al., 2017; Akkermans et al., 2018). In spite of this, further investigation is required to delineate the relationship between musical training and recognition of non-verbal emotional expression.

Given that superior emotion recognition ability could result from enhanced acoustical processing, it follows that fundamental differences in auditory perception may also influence recognition ability. The pitch and duration of musical events are important cues for interpreting emotional expression in both speech and music (Juslin and Laukka, 2003; Lima et al., 2016), meaning that differences in perceptual sensitivity may be predictive of differences in emotion perceived in music. This hypothesis is reinforced by studies of individuals with hearing impairments, who show deficits in processing of emotion in both music and speech which align with difficulties processing pitch (Wang et al., 2013) and timbral variations such as roughness (Paquette et al., 2018).

The current research was inspired by a recent replication and extension (Akkermans et al., 2018) of a study carried out by Gabrielsson and Juslin (1996). The original study investigated communication of emotion in music using a production-recognition paradigm. Firstly, professional musicians (including flautist, guitarists, violinists, and vocalists) were asked to perform three melodies several times; for each performance they were instructed to adjust their expressive intentions to convey a specific emotion (happy, sad, angry, fearful, tender, solemn, or without expression). Performance recordings were analyzed in terms of their musical and acoustic properties to identify the expressive cues characteristic of each emotion. These recordings were then used for listening experiments, in which participants were asked to identify performer-intended expressions. Results indicated that the performers’ intentions were mostly identified correctly, with a higher decoding accuracy for basic emotions, in accordance with Juslin’s (1995) hypothesis regarding the comparative ease of communicating basic versus complex emotions. In the replication study, emotional and musical individual differences were assessed with regard to their influence on emotion-decoding ability (Akkermans et al., 2018). Participants’ ability to accurately decode musical emotions was found to be associated with their level of musical training.

The main objectives for the current study were: firstly, to develop a short and effective Musical Emotion Discrimination Task (MEDT), which tests an individuals’ ability to decode emotions in music using a simple response format. Secondly, to examine individual differences in EI, EC, musical training, and emotional engagement in relation to their influence on perceived emotion in music, and finally, to extend previous research by investigating the contribution of low-level auditory ability to emotion decoding performance. Three experiments were carried out. Experiment 1 consisted of a preliminary MEDT, in which two excerpts of the same melody were presented per trial, which differed only in terms of emotional expression conveyed through the performance. Excerpts differed between trials in terms of musical features such as length, instrument, melody, target emotion and comparison emotion. Item difficulty was assessed with regard to the contribution of these features, with the expectation that they would affect task performance as found previously (Akkermans et al., 2018). Furthermore, this analysis informed a shorter test of emotion discrimination by allowing for the calibration of overall test difficulty. The short MEDT was then tested and further refined in experiment 2. The test was employed alongside other measures of relevant abilities to allow for a preliminary assessment of test validity. It was hypothesized that participants’ superior emotional, musical and perceptual abilities, would coincide with a superior ability to decode performer-intended emotions. Experiment 3 was conducted in order to further establish the usefulness of the test as a measure of musical emotion decoding by investigating the overlap between the MEDT and measures of general emotion abilities such as emotion recognition from facial and vocal stimuli, and of emotion deficits such as alexithymia. Accordingly, it was expected that performance on general emotion recognition tasks would be positively linked with MEDT performance, while self-reported levels of alexithymia would be negatively related to performance.

**FIGURE 1:**
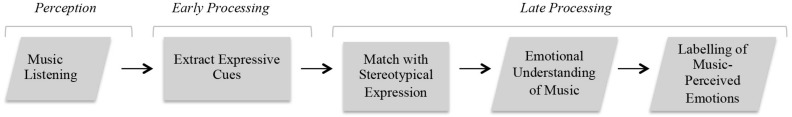
A diagram to illustrate the cognitive model proposed to underlie emotion recognition in music as relevant to the testing paradigm of the MEDT. The rectangles reflect covert processes that cannot easily be directly measured or controlled, while the parallelograms represent processes that can be manipulated and studied.

To better understand how such individual differences might impact on emotion decoding, it is useful to view them as part of a cognitive process model. The following therefore describes a simple model that can be used to understand the processes underlying the decoding of music-expressed emotions (see Figure 1), which can be used to account for the influence of other relevant cognitive abilities. At the first stage, a listener must perceive an external musical stimulus and extract expressive auditory cues such as tempo, articulation, or dynamics. Next, the listener must meaningfully identify these cues by matching them to stereotypical expressions of musical emotion. This process is thought to rely on general emotion processing mechanisms responsible for the understanding of emotional sounds, such as those engaged within the processing of speech prosody (Juslin and Laukka, 2003), as well as on schemas built through previous music listening or music performance experience. Finally, the listener can use the information gained from these cues through the matching process to facilitate an emotional understanding of the stimulus. For example, an individual may listen to a musical piece with a slow tempo and (subconsciously) extract this as an expressive cue. Due to previous associations with sad music and the potential overlap with characteristics of sad vocal expression, this feature may be linked with a stereotypical expression of sadness and could therefore cause them to identify the piece as sad.

This model is informed by current literature exploring the extent to which processes involved in the perception and interpretation of acoustic cues of emotion in speech and music are shared. There are numerous examples of overlaps between emotional cues used in speech and music (Juslin and Laukka, 2003). For instance, Curtis and Bharucha (2010) provide an analysis of vocal portrayals of emotion performed by American actors, which reveals the prominence of minor third intervals in portrayals of sadness theorized to occur as a result of physiological effects of emotion. Interestingly, minor third intervals are commonly interpreted to represent sad expression in music. Cross-cultural research has also demonstrated significant cross overs between emotional cues in both eastern and western music and vocal expressions, displaying that positive emotions are conveyed using large melodic or prosodic intervals compared to smaller intervals used to convey negative emotions (Bowling et al., 2012). Though these findings relate exclusively to melodic intervals, which are controlled in the current study through use of the same melody between emotions, there is also strong evidence for the impact of emotional properties such as rate and intensity on the processing of speech and music, where fast paced, loud speech is interpreted to be similar in valence to fast paced, loud music (for example, Ilie and Thompson, 2006). Such evidence reinforces earlier ideas put forward by Juslin (1997) within the functionalist perspective which stipulate that similarities between vocal and musical expression rely on shared communicative systems within the brain which are present from birth and are strengthened through social interaction. The present model thus endorses the possibility of shared processing as hypothesized by Juslin (1997), and the following investigation aims to determine whether this is mirrored by positive correlations between performance on musical and vocal emotion recognition tasks.

**FIGURE 2:**
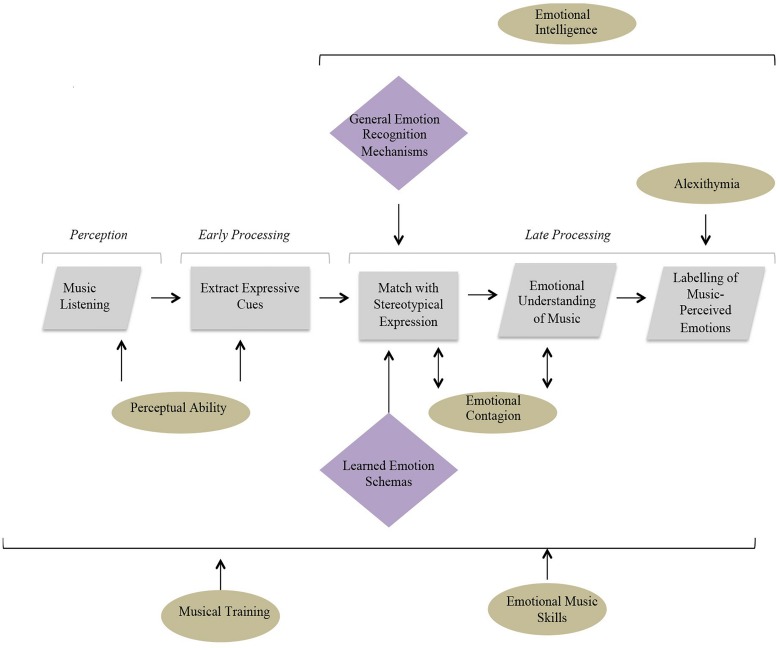
A diagram displaying the contribution of individual differences (in circles) at different stages of a cognitive model proposed to underlie emotion recognition in music. The diamond shapes highlighted in purple represent cognitive mechanisms thought to underlie the operation of particular processes.

It is speculated that specific cognitive abilities may only play a role at certain processing stages within the proposed model. For example, perceptual ability can only influence early auditory processing, whereas EI is likely to have more impact at later stages involving more general emotion mechanisms responsible for the processing of vocal and facial emotions. Emotional contagion and alexithymia are also higher-level processes, involved in later cognition, although their effect may be more restricted to individual processing stages. For example, alexithymia, a condition associated with impairments in verbal formulation of emotion (Taruffi et al., 2017) is expected to only impact upon the final phase involving labeling of music perceived emotions. Musical training, on the other hand, has the potential to impact all stages of processing. Previous research has demonstrated the effect of training on both the perception of music (Musacchia et al., 2007; Kraus and Chandrasekaran, 2010) as well as on higher-level processes such as emotion decoding (Akkermans et al., 2018). The model below illustrates how individual differences are hypothesized to impact upon different stages of processing and therefore provides a starting point for the following investigation (see Figure 2).

## Experiment 1

### Method

#### Participants

Seventy seven participants were recruited online via social network platforms and the Goldsmith’s research participation scheme; only those recruited through the research scheme received compensation, which was administered in the form of course credit. Participants ranged from 18 to 80 years of age (M = 37.06, SD = 22.65), and included 26 females, 18 males, and 2 individuals who preferred to withhold gender information and 31 who did not provide demographic information. The current study was granted ethical approval by Goldsmith’s Research Ethics Committee.

#### Materials

##### Stimuli recording

**FIGURE 3:**
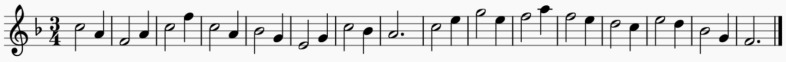
Notation of melody B (1).

**FIGURE 4:**
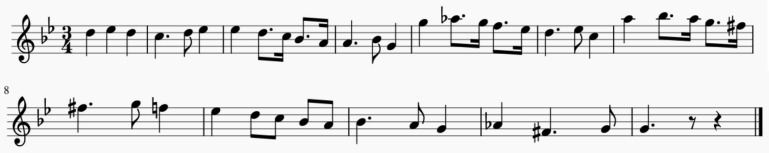
Notation of melody C (2).

For the MEDT, melodies B and C from Gabrielsson and Juslin’s (1996) study were employed. Melody B is a Swedish folk melody in F major which spans a two-octave range and is entirely diatonic (see Figure 3), while Melody C was composed specifically for use within their research. Melody C is in G harmonic minor, spans two octaves and contains a few chromatic notes (see Figure 4).

Hereafter, melody B will be referred to as melody 1, and melody C as melody 2. The musical extracts utilized in the current study were re-recordings of the stimuli first validated by Gabrielsson and Juslin (1996). The replication study carried out by Akkermans et al. (2018) validated the re-recorded versions of the stimuli. In the present study, only recordings that conveyed angry, happy, sad, and tender expressions on piano, violin, or voice were used, as findings indicated these tended to be identified most accurately by listeners (Akkermans et al., 2018). On average, angry excerpts were 15s long, while happy excerpts were 16s, sad excerpts were 35s, and tender excerpts were 31s. Duration and tempo for each of the melodies selected for the current experiment is provided in Table 1. Tempo was estimated manually by extracting the average beats per minute (bpm) across the entire clip to account for the performers’ use of rubato.

**TABLE 1:** Stimulus properties of the melodies from Akkermans et al. (2018) employed in the current study.

**Instrument**	**Emotion**	**Melody**	**Average tempo (bpm)**	**Duration (s)**
Pi	A	B	205	16
Pi	A	C	132	19
Pi	H	B	215	16
Pi	H	C	117	19
Pi	S	B	93	32
Pi	S	C	54	39
Pi	T	B	96	32
Pi	T	C	51	41
Vi	A	B	343	9
Vi	A	C	191	12
Vi	H	B	292	11
Vi	H	C	167	14
Vi	S	B	84	34
Vi	S	C	88	25
Vi	T	B	113	26
Vi	T	C	121	17
Vx	A	B	225	15
Vx	A	C	117	20
Vx	H	B	179	18
Vx	H	C	125	18
Vx	S	B	109	38
Vx	S	C	64	40
Vx	T	B	97	36
Vx	T	C	65	34

*“Pi” stands for piano, “Vi” stands for violin, and “Vx” stands for voice, while “A” stands for anger, “H” stands for happy, “S” stands for sad, and “T” stands for tender. “B” represents melody B, the Swedish folk melody and “C” represents melody C, a melody composed specifically for use in the research conducted by Gabrielsson and Juslin (1996).*

Acoustic analyses of these clips were carried out by Akkermans et al. (2018), who found that emotions tended to possess distinct acoustic features; the key features of angry excerpts were high amplitude, fast tempo and greater roughness, happy excerpts were most similar to angry excerpts though did not display such high roughness, sad excerpts exhibited slow tempos and low amplitude, and tender excerpts displayed similar acoustic properties as those conveying sadness. For further detail on the acoustic properties of the stimuli readers are referred to the paper by Akkermans et al. (2018) which provides a comprehensive analysis.

##### Stimuli editing

Recordings were edited in order to establish a greater variation in terms of difficulty. This was achieved by splitting audio files into musically meaningful phrases using Adobe audition CC. Melody 1 was split into four 4-bar phrases, while melody 2 was split into six 2-bar phrases. Subsequently, audio files were produced from all possible combinations of consecutive phrase sequences. For example, one clip of melody 1 was edited to produce 10 separate clips: four one-phrase clips, three two-phrase clips (e.g., 1 and 2, 2 and 3, and 3 and 4), and three three-phrase clips (e.g., 1, 2 and 3, and 2, 3, and 4). Excerpts were then paired, each pair featuring the same combination of musical phrases played on the same instrument by the same performer but two contrasting performances aiming to convey distinct emotions. These pairs were combined into a single mp3 file using SoX (sound exchange) software^1^, with a buzzer sound inserted in-between. Thus, 1116 items were produced that featured two clips with the same melody, instrument, and phrases, but differing emotional expressions.

###### Musical emotion discrimination task

The MEDT initially consisted of 112 items, selected to represent the larger corpus of 1116 items. These were selected at random, under the condition that each musical feature under assessment must be equally represented, for example half of the extracts were melody 1 and half melody 2. Correspondingly, a third were played on the piano, a third on the violin and a third sung. From the pool of 112 selected test items, 21 were randomly presented to each participant. Responses were collected using a two-alternative forced choice (2-AFC) format.

##### Depression screening

The Patient Health Questionnaire (PHQ-9), a short, self-administered survey, was used to assess depression severity (Kroenke and Spitzer, 2002). This measure consists of nine items related directly to the DSM-IV diagnostic criteria.

#### Procedure

This experiment was conducted online which allowed for automatic administration of the information sheet, consent form, depression screening, MEDT, demographics form, and debrief. For the MEDT, participants were told that for each trial, they would hear two versions of a melody that would differ in terms of emotional expression. They were instructed to indicate which version they felt was most representative of a given emotion. Each participant was exposed to 21 audio clips and instructed as follows: “Please listen to the following clips and select which one sounds happier to you. Select 1 for the clip heard before the buzzer, or 2 for the clip heard after the buzzer.” The attribute happier would be exchanged for the target emotion of the item. The task took around 15–20 min to complete.

### Results

From the initial sample of 77 participants, 34 participants were excluded from analysis, as they had not fully completed the experiment. Additionally, 10 participants were excluded as their scores were above the typical cut off point (≥10) in the depression screening (Manea et al., 2012), on account of the previous finding that depressed individuals display difficulties processing emotions in music (Punkanen et al., 2011).

#### Musical Features

Mixed-effects logistic regression analyses were performed to determine whether the stimulus features length and target emotion influence the correctness of participant responses and to account for random effects resulting from differences between participant abilities. Binary item responses (0 = incorrect and 1 = correct) served as dependent variable, target emotion and length were treated as predictors and participant ID was used as random factor. The binomial mixed effects model (i.e., logistic regression models) including only the random factor was compared against two models that contained length or target emotion as predictors. Likelihood ratio tests indicated that each predictor contributed to the accuracy of the model beyond the random factor (for model including length *p* = 0.011, for model including target emotion *p* = 0.002). The significant contribution of both length and target emotion to participant accuracy was confirmed by type II Wald Chi-square tests on the predictors of the final model including target emotion (χ^2^(3) = 13.6, *p* = 0.003) and length (χ^2^(1) = 5.6, *p* = 0.018). As expected, length showed a significant positive effect on item easiness. This means that longer items generated more correct responses. Target emotion also affected item difficulty/easiness with happy and tender items being significantly more difficult than angry items (*p* = 0.012 and *p* = 0.011) which served as the reference category. In contrast sad items did not affect item difficulty/easiness compared to angry items (*p* = 0.764).

### Discussion

The focus of experiment 1 was to determine whether variation of musical features affected item difficulty. Two features of significance were: extract length (i.e., number of phrases featured in the audio clip), and target emotion (i.e., the emotion expressed within the extract that participants were asked to identify). Excerpts featuring happy or tender as a target emotion were more difficult than angry items, and shorter items were more difficult than longer items, according to logistic regression models.

These findings were used to inform a shorter test of emotion discrimination ability for use in experiment 2. It is likely that shorter excerpts were harder to discriminate owing to the fact they contain fewer expressive cues, thus only items featuring only one phrase of the melody were retained in order to adjust the overall level of correct responses from 83.4 to 75%, halfway between optimal and chance performance. Items containing two or three melodic phrases were eliminated. As a result, there were few items remaining that featured melody 2, owing to the initial stimuli selection process. Results indicated that melody 1 had a lower overall percentage of correct responses than melody 2; hence, all items featuring melody 2 were removed. Despite finding that item difficulty was influenced by target emotion, this variable was not used as criteria for item elimination in order to maintain the range of possible target and comparison emotions across the test. Therefore, the shortened test was comprised of 28 items, which differed in terms of target emotion, comparison emotion and instrument.

## Experiment 2

### Method

#### Participants

One hundred and two participants (64% female, 32% male, 1% “other,” and 3% with no gender information), were recruited via the Goldsmiths research participation scheme, as well as through social media and poster advertisements. Four of these had also taken part in experiment 1. Most were undergraduate students. Other than those recruited via the research scheme, who received course credit in exchange for participation, participants did not receive any compensation. Participants ranged from 18 to 52 years of age (M = 24.11, SD = 6.26). In total, 25 participants (54% female) took part in the re-test, which was conducted at least a week after the first test session, ranging from 21 to 75 years of age (M = 28.3, SD = 12.68). Fifteen were recruited from the initial sample (*N* = 102), and nine participants were recruited at a later stage. This study gained ethical approval from Goldsmiths Research Ethics Committee.

#### Materials

##### Individual difference questionnaires

The Goldsmiths Musical Sophistication Index (GOLD-MSI), was used to assess musical behaviors using a self-report questionnaire (Müllensiefen et al., 2013). This inventory consists of five sub-scales, of which three were used to measure musical training (MT) as well as emotional music skills (EMS) and active engagement (AE) with music.

The Trait Emotional Intelligence Questionnaire Short Form (TEIQue-SF) was administered to measure EI via self-report (Petrides, 2009).

Emotional contagion was evaluated using the Emotional Contagion Scale (Doherty, 1997), which consists of 15 self-report items, including hypothetical scenarios such as “When someone smiles warmly at me, I smile back and feel warm inside.”

##### Musical emotion discrimination task

The 28-item version of the test described in the results section of experiment 1 was employed for experiment 2.

##### Perceptual discrimination tasks

Psychoacoustic tests were employed to establish participants’ ability to discriminate duration and pitch. These were run using two experiments from the Maximum Likelihood Procedure (MLP) toolbox on MATLAB 2013b (Grassi and Soranzo, 2009): pitch discrimination complex tone and duration discrimination complex tone. Experiments were set up so that two blocks of 20 trials were completed per test, and responses were collected using a 3-AFC format. An auditory threshold estimate was produced for each block of trials and the lower of the two thresholds was retained for analysis. Default settings, as specified by the MLP toolbox, were otherwise maintained. Participants carried out both the new MEDT and psychoacoustic tests using either AKG-K451 or Behringer HPM1000 headphones and responses were collected using a computer keyboard and mouse.

#### Procedure

For this experiment, the MEDT and psychoacoustic tests were completed in a silent, controlled setting. The four participants who had taken part in experiment 1 completed these tests 1–3 months after their initial testing session. Participants who had not taken part in experiment 1 were asked to complete the individual difference questionnaires online, either before or after the in-lab tests took place. At the beginning of each part of this study, participants were provided with an information sheet and consent form.

For the short MEDT, participants received the same instructions as were provided in the first experiment; this task took approximately 8–10 min. Following this, participants took part in two psychoacoustic tests; for each test, they were told that they would hear three tones per trial. For the first, they were asked to distinguish which tone was longer in duration, while for the second they were asked to identify which was higher in pitch. Each test took around 3 min to complete.

### Results

Three participants were excluded from analysis, as they had not completed the individual difference questionnaires, leaving 99 cases. Data from participants who had scored above the typical cut-off point (≥10) on the depression screening (Manea et al., 2012) was retained for analysis, on the basis that there was no significant correlation between depression scores and MEDT performance in experiments 1 and 2.

#### Multiple Regression Analyses

A multiple regression was performed to establish whether EI, EC, MT, EMS, pitch discrimination, and duration discrimination predicted MEDT performance. Depression scores were also included as a predictor in this analysis. The active engagement variable was excluded from further analysis as it was highly correlated with emotional engagement (*r*(99) = 0.76, *p* < 0.001, two-tailed). The overall model was significant *R*^2^ = 0.14, adjusted *R*^2^ = 0.08, *F*(7, 91) = 2.14, *p* = 0.047, though none of the seven predictors contributed significantly to the model (see Table 2).

**TABLE 2:** Regression model with MEDT scores as dependent variable (*N* = 99).

	**B**	**SE**	**β**	***p***
Constant	24.04	12.82		0.06
Emotional intelligence (EI)	0.44	0.35	0.17	0.21
Emotional contagion (EC)	0.02	0.02	0.1	0.36
Musical training (MT)	0.013	0.02	0.07	0.53
Emotional music skills (EMS)	0.06	1.38	0.17	0.14
Pitch discrimination	0	0.04	–0.03	0.79
Duration discrimination	–0.02	0.01	–0.12	0.23
Depression scores	0	0.05	0	0.97

*B represents the regression coefficient and β represents the standardized coefficient. SE, standard error.*

In a second analysis, backward elimination was used to discard variables that were not significantly contributing to the model (*p* < 0.05). The final model using MEDT sum scores as dependent variable indicated that EI and EMS significantly predicted MEDT performance, *R*^2^ = 0.12, adjusted *R*^2^ = 0.1, *F* (2, 96) = 6.39, *p* = 0.002, as outlined in Table 3.

**TABLE 3:** Regression model with MEDT sum scores as dependent variable using backward elimination of predictor variables.

	**B**	**SE**	**β**	***p***
Constant	16.28	1.49		<0.001
Emotional intelligence (EI)	0.5	0.26	0.19	0.06
Emotional music skills (EMS)	0.09	0.04	0.23	0.03

In addition, correlational analyses were used to assess whether individual difference and perceptual measures were associated with the MEDT scores, which were computed for each participant using a sum score (see Table 4 for descriptive statistics). One-tailed tests, using Bonferroni correction for multiple comparisons (*p* = 0.007), revealed that EI and EMS were significantly and positively correlated with performance on the MEDT (see Table 5). However, MEDT performance was not significantly associated with EC, MT, AE, or pitch and duration discrimination tests.

**TABLE 4:** Descriptive statistics from experiment 2 (*N* = 99).

	**M**	**SD**	**Range**
MEDT score	21.55	2	17–26
Emotional intelligence (EI)	4.8	0.77	2.8–6.3
Emotional contagion (EC)	50.24	9.11	29–70
Musical training (MT)	21.16	10.69	7–46
Active engagement (AE)	38.94	12.06	10–62
Emotional music skills (EMS)	33.27	5.43	14–42
Pitch discrimination	335.03	5.84	330.76–365.31 Hz
Duration discrimination	279	13.81	256.85–330.03 ms

*MEDT, musical emotion discrimination test.*

**TABLE 5:** Matrix displaying Pearson’s *r* correlations (one-tailed, *p* = 0.007, Bonferroni corrected) between MEDT score and individual difference measures (*N* = 99).

	**MEDT score**	**EI**	**EC**	**MT**	**AE**	**EMS**	**Pitch**
MEDT score	−						
EI	0.26^∗^	−					
EC	0.2	0.34^∗^	−				
MT	0.19	0.13	0.19	−			
AE	0.22	0.18	0.27	0.58^∗∗^	−		
EMS	0.28^∗^	0.31^∗^	0.32^∗^	0.41^∗∗^	0.76^∗∗^	−	
Pitch	–0.14	–0.14	–0.09	−0.26^∗^	−0.3^∗^	−0.27^∗^	−
Duration	–0.08	0.03	0.21	–0.001	0.029	0.09	0.07

*EI, emotional intelligence; EC, emotional contagion; MT, musical training; AE, active engagement; EMS, emotional music skills. Pitch and Duration represent perceptual discrimination thresholds, for which lower values represent better performance. ^∗^*p* < 0.007, ^∗∗^*p* < 0.001.*

#### Final Item Selection

From the set of 28 items, we removed three of the items that all 99 participants had responded to correctly. On the remaining 25 items we performed an item response theory (IRT) analysis with the aim of obtaining a sound measurement model of emotion discrimination ability as well as reducing the overall test length by eliminating items that would not fit the IRT model framework.

Given the moderate size of our sample we followed the advice given by de Ayala (2009) and aimed to avoid model overfitting. Hence, we constructed a simple Rasch model which requires only the estimation of item difficulty parameters. Using the R package mirt (Chalmers, 2012) we computed an initial Rasch model with item difficulty parameters and fixing the discrimination parameter to 1. Because the experimental task was a two-alternative forced choice task, we also included a guessing parameter into the model constrained to be equal across all items and fixed the guessing value to 0.4, allowing for a small bias toward the wrong response option. The total test information of the 25-item model over the difficulty range from -4 to 4 was 5.92. In a second step we excluded six items which showed a moderate to severe bias toward the wrong response option, having an overall percentage correct that was significantly lower than the 50% chance level (i.e., percentage correct < 40%, as determined by a two-sided binomial test with alpha level at 0.05 and 99 participants). This 19-item model had an identical test information value of 5.92 (after rounding to two decimals).

Inspecting the item-total score correlations we identified one item with a negative correlation coefficient which was subsequently eliminated. This third model comprised 18 items and the total test information was still 5.37 and hence showed only minor decrease in test information compared to the 25-item model. The 18-item model was accepted as the final model. It showed no indication of model misfit (M2 = 150, df = 152, *p* = 0.53). The mean-square infit statistics of all 18 items and the outfit statistics of 12 items were in the range between 0.5 and 1.5, which is deemed “productive for measurement” (Linacre and Wright, 1994). The outfit statistics of six items were <0.5, which is considered “less productive for measurement but not degrading” (Linacre and Wright, 1994). The model’s test-retest reliability correlation was at 0.69 (*N* = 24) and model’s empirical reliability based on the person ability estimates and standard errors computed with weighted likelihood estimation was 0.75 (*N* = 99). The 18-item test has since been implemented in R shiny package (Chang et al., 2018) and is available for research use upon request.

### Discussion

The purpose of experiment 2 was to evaluate the suitability of the refined test to the assessment of musical emotion decoding. The suitable calibration level of the 28-item test was established on the basis that average task performance was approximately halfway (77%) between perfect performance and chance level on the 2AFC task. Furthermore, experiment 2 allowed for the investigation of factors influencing the ability to discriminate performer-intended expressions of emotion in music. It was expected that those with superior emotional, musical and perceptual capabilities would exhibit superior discrimination. The multiple regression analyses including seven individual difference factors indicated that only a small proportion of the variance in MEDT scores were explained, thus refuting our initial hypotheses. Correlational analyses provided no evidence to suggest that musical expertise or heightened perceptual acuity (i.e., pitch and duration discrimination ability) was related to MEDT performance, though they did indicate that those who were more emotionally skilled appeared to hold an advantage.

The correlations between MEDT scores and EI and EMS back up previous studies which have linked individual differences in general emotional capabilities to individual differences in emotion recognition ability in the music domain (Resnicow et al., 2004; Akkermans et al., 2018) and thus provide evidence for the hypothesized overlap between general emotion mechanisms and those involved in processing musical emotion. However, when these factors were included in a multiple regression model alongside five other predictors, they failed to demonstrate a significant contribution to MEDT scores. This may be in part due to the variance that EI and EMS share with other predictor variables in the full regression model. Consequently, a reduced regression model resulting from backward variable selection did show significant associations of EI and EMS with MEDT scores. In contrast, emotional contagion, considered to be a key facet of EI (Salovey and Mayer, 1990) and hence a vital component of general emotion processing did not contribute significantly to any of the regression models and was not significantly correlated with MEDT performance. Overall, this demonstrates a weak link between general emotion mechanisms on musical emotion decoding ability, contrary to expectations.

Formal musical training also demonstrated no additional effect on the decoding of expressive performance, which corresponds with the negative findings from Trimmer and Cuddy (2008), but directly contradicts positive reports from the experiment conducted by Akkermans et al. (2018) and previous research from Lima and Castro (2011). While this result therefore contradicts our hypotheses and contributes little to the debate regarding impact of musical expertise on musical emotion perception, it can be argued that this demonstrates the current test measures a subset of skills related exclusively to the processing of musical emotions, as opposed to musical skills more generally. It is also possible that musical training may indirectly affect processes involved in emotion decoding. The correlations outlined in Table 5 demonstrate that MT contributes to EMS, but not to MEDT scores, while EMS are related to both. Thus, in the presence of EMS, MEDT scores can be predicted without MT scores, though MT may still play a role due to its connection with EMS. This possibility must be confirmed in future studies using more stringent hypothesis testing.

Following from the non-significant contribution of musical training to musical emotion decoding, it may be considered unsurprising that superior processing of perceptual cues of pitch and duration does not translate to a more sophisticated interpretation of auditory emotional expression, as reflected by the negligible effect of perceptual thresholds on MEDT performance. It has been postulated that the perception of musical emotion may be based on the interpretation of a complex combination of acoustic cues, and not depend simply on individual low-level acoustic features (Filipic et al., 2010). Accordingly, Filipic et al. (2010) found that emotion judgments were not influenced by the variation of discrete perceptual features such as dissonance and loudness, which is in line with the non-significant correlations between psychoacoustic discrimination abilities and performance on the MEDT in Exp. 2. In contrast, results also displayed a relationship between pitch discrimination and EMS, MT, and AE (see Table 4), highlighting that perceptual features may in fact contribute to higher level emotion processing of acoustic material, though they did not contribute to performance in the MEDT.

Also relevant to this finding is evidence of preserved musical and vocal emotion processing in individuals with amusia, a condition which affects the processing of discrete musical elements such as pitch (Gosselin et al., 2015). One could speculate that this dissociation might also apply to healthy individuals, and thus that pitch processing may not be vital for emotion decoding, as indicated by the current results. Further research into the level of overlap between low-level auditory and high-level emotion perception within the healthy population is necessary to establish the extent to which perception of auditory cues impacts emotion processing.

An alternate explanation for the low correlations is the fact that the current sample did not exhibit much variation in perceptual ability; this could be due to the limited age range typical of recruitment from student populations. As auditory processing capabilities are generally found to be associated with age (Tun et al., 2012), it is possible that the predominance of younger participants contributed to a lack of meaningful differences in psychoacoustic ability. A sample which more closely represents the age range of the general population, in which differences in auditory perception are likely to be more pronounced, would therefore be more suitable to test for an effect of pitch and duration processing on emotion discrimination ability.

Another issue worth highlighting is the relatively small sample recruited for an individual differences study like the current experiment. It is likely that the limited explanatory power of the multiple regression model is partially attributable to the fact that the current sample was of insufficient size to detect subtle variations within the numerous measures of individual differences examined in this study, as investigations of individual differences require large samples in order to detect such small effects (Gignac and Szodorai, 2016). While the backward elimination model extracted EI and EMS as significant predictors of MEDT score, it is important to note that such data-driven techniques bear the danger of overfitting and thus opportunities for the interpretation and generalization of the current findings are limited. The findings of the current study therefore require validation with a larger sample to ensure the statistical power necessary to detect the predicted effects.

## Experiment 3

The aim of experiment 3 was the assessment of the convergent and divergent validity of the new 18-item emotion discrimination test, comparing performance on the new test to performance on other established measures related to emotion processing and musical expertise. This included two measures of visual and auditory processing, one self-report inventory for emotion naming ability and a multidimensional self-reporting inventory assessing musical expertise and behaviors.

### Method

#### Participants

150 participants (81% female, 18% male, and 1% with no gender information), were recruited from among Goldsmiths undergraduate psychology students and received course credit in exchange for participation. Participants ranged from 18 to 36 years of age (M = 19.38, SD = 3.02). None of those recruited had taken part in either of the previous experiments. This study gained ethical approval from Goldsmiths Research Ethics Committee.

#### Materials

The short 18-item version of the MEDT described in Experiment 2 was employed in Experiment 3. A complete outline of the instruments, emotions, duration, tempo and difficulty of these items along with their IRT item difficulty estimates is provided in Table 6. The ability to process visual as well as non-musical auditory emotional stimuli was assessed through the Emotion Recognition Index (ERI; Scherer and Scherer, 2011) tests. The ability to verbalize emotions was assessed via the alexithymia screening measure TAS-20 (Bagby et al., 1994) and as in Experiment 2 the GOLD-MSI (Müllensiefen et al., 2014), was used to assess musical expertise and behaviors. The focus was on the correlations between MEDT and the Musical Training and Emotions subscales of the Gold-MSI as indicators of convergent validity.

**TABLE 6:** Stimulus properties of the 18 items featured in the final version of the MEDT.

	**Target stimulus**		**Comparison stimulus**			
**Instrument**	**Emotion**	**Tempo (bpm)**	**Emotion**	**Tempo (bpm)**	**Duration (s)**	**Item difficulty**
Pi	A	205	H	215	10	0.2
Pi	A	205	S	93	16	3.05
Pi	A	205	T	96	16	2.77
Pi	H	215	A	205	10	–3.16
Pi	H	215	S	93	14	4.41
Pi	H	215	T	96	15	3.05
Pi	T	96	H	215	16	3
Vi	A	343	H	292	8	1.99
Vi	A	343	T	113	12	2.48
Vi	H	292	T	113	13	4.43
Vi	S	84	A	343	15	5.3
Vi	T	113	H	292	12	3.38
Vx	A	225	T	97	15	5.23
Vx	H	179	S	109	15	2.87
Vx	H	179	S	109	17	3.4
Vx	H	179	T	97	18	2.39
Vx	H	179	T	97	16	1.88
Vx	H	179	A	225	10	4.48

*Tempo was calculated manually prior to editing (refer to section “Experiment 1 – Materials”) and thus serves as an estimate. “Pi” stands for piano, “Vi” stands for violin, and “Vx” stands for voice, while “A” stands for anger, “H” stands for happy, “S” stands for sad, and “T” stands for tender, “bpm” stands for beats per minute, “s” stands for seconds.*

#### Procedure

All tests and self-report measures were administered via a browser-based online interface. Participants were tested in groups in a lecture theater and used their own wireless devices to access the experiment’s starting page that contained information sheet and consent form as well as links for the individual tests and self-report inventories. Participants chose the order of the tasks themselves and completed the tasks at their own pace. Technical difficulties arising from the interaction of certain devices, operating systems, the media server, and the university firewall meant that a few participants were not able to see the images and sounds of the ERI, which resulted in missing values for those participants on these tasks and are responsible for the varying sample sizes reported in the results below.

### Results

For the MEDT, accuracy scores were computed for all participants by summing correct responses and converting to percentages (see Table 7 for descriptive statistics). In addition, IRT scores were computed from the IRT model described in Experiment 2 using the Bayes Modal scoring method. As Table 8 shows, the correlation between the accuracy and model-based MEDT scores is very high (*r* = 0.96) and the pattern of correlations between the two MEDT scores and the five measures of emotion processing and musical expertise is very similar.

**TABLE 7:** Descriptive statistics from experiment 3.

	**M**	**SD**	**Range**
Accuracy score (*N* = 104)	83.4	0.12	44–100
IRT score (*N* = 104)	0.02	0.54	−1.46–1.57
Alexithymia (*N* = 107)	50.06	10.53	23–77
Facial recognition (*N* = 53)	65.66	10.91	20–87
Vocal recognition (*N* = 53)	60.51	9.77	37–80
Musical training (*N* = 140)	18.59	9	7–44
Emotional music skills (*N* = 140)	31.45	5.33	13–42

**TABLE 8:** Matrix displaying Pearson’s *r* correlations (one-tailed, *p* = 0.008, Bonferroni corrected) between the two MEDT scores and the five measures of emotion processing ability and musical expertise.

	**Accuracy score**	**IRT score**	**TAS-20**	**Facial**	**Vocal**	**MT**
Accuracy score	−					
IRT score	0.8^∗∗^(150)	−				
Alexithymia	−0.07(107)	−0.02(107)	−			
Facial recognition	0.44^∗∗^(53)	0.32(53)	−0.22(51)	−		
Vocal recognition	0.21(53)	0.13(53)	−0.3(51)	0.43^∗^(53)	–	
Musical training (MT)	0.11(140)	0.15(140)	0.05(103)	−0.07(52)	−0.17 (52)	
Emotional music skills (EMS)	0.33^∗∗^(140)	0.33^∗∗^(140)	−0.22(103)	−0.05(52)	0.29 (52)	0.36^∗∗^ (144)

**r* represents Pearson’s correlation coefficient. *N* displayed in brackets. ^∗^*p* < 0.008, ^∗∗^*p* < 0.001.*

Two multiple regression models were constructed to predict MEDT accuracy scores and IRT model-based scores using measures of alexithymia, facial recognition, vocal recognition, MT and EMS as predictors. The lavaan package in R (version 0.6-3) was employed for modeling because of the option to account for missing data using the finite information maximum likelihood estimation method (FIML). The regression models (*N* = 150) revealed that facial recognition and EMS scores explained a significant amount of variance in both models, using MEDT accuracy (see Table 9) and IRT scores. However, amount of variance explained was higher for the regression model using accuracy scores as dependent variable (*R*^2^ = 0.42) compared to the model that used IRT scores (*R*^2^ = 0.3), even though the set of predictor variables in both models was identical.

**TABLE 9:** Regression model with MEDT accuracy scores as dependent variable (*N* = 150).

	**B**	**SE**	**β**	***z***	***p***
Constant	0.17	0.12	1.42	1.41	0.16
Alexithymia	0	0	0.12	1.17	0.24
Facial recognition	0.01	0	0.59	5.34	<0.001
Vocal recognition	0	0	−0.07	−0.43	0.67
Musical training (MT)	0	0	0.01	0.147	0.88
Emotional music skills (EMS)	0.01	0	0.36	3.3	0.001

*B represents the regression coefficient, β represents the standardized coefficient, and *z* represents a test statistic. SE, standard error.*

As expected, the MEDT scores correlate positively and significantly with the ability to process visual emotional stimuli as well as with self-reported ability to handle musical emotions in a sophisticated way. In contrast, the correlation between the MEDT scores and MT is substantially lower, indicating that MEDT scores are not just another measure of formal musical training but measure a specific musical ability. The non-significant correlation with the TAS-20 suggests that the ability to name the perceived emotion is not a crucial part of the psychological processes required for solving the MEDT. This seems plausible given that the emotional attribute is explicitly named in the prompt of each trial and no independent emotion naming is required from the participants. Finally, the relatively low correlation between the MEDT performance and the ability to decode vocal emotional stimuli is surprising, given that both tasks require the extraction of cues from auditory input. Refer to Table 8 for the full correlation matrix.

### Discussion

**FIGURE 5:**
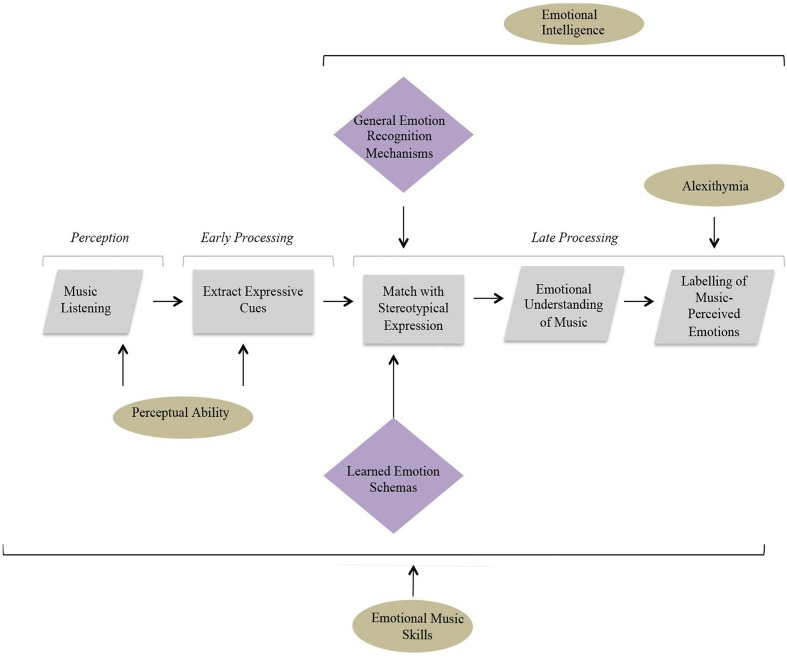
An illustrative model of musical emotion decoding informed by the results of the current study.

Findings from the first experiment contributed to the development of a shorter measure of emotion discrimination implemented within experiments 2 and 3. These results attest to the validity of the short 18-item MEDT as a measure of musical and emotional processing and help clarify the relative importance of different abilities within the linear model of musical emotion discrimination proposed at the outset of the investigation (refer to Figure 2). Illustrated in Figure 5 is an updated model of understanding and decoding emotions in music informed by the current study. This model represents a general account of musical emotion perception, not limited to the emotional processes employed during the MEDT, hence the inclusion of emotion labeling at the final stage.

The fundamental question addressed within the current study was whether general emotion mechanisms are crucially involved in musical emotion decoding. The significant contribution of the emotions subscale of the GOLD-MSI to MEDT performance provides evidence for the involvement of processes specific to the translation of emotion conveyed through music, and bolsters the predictive validity of the MEDT. These results support recent findings (Akkermans et al., 2018) and seem intuitive considering that those who listen to and engage with music emotionally on a regular basis are likely to be more familiar with typical expressive cues. It is thus clear that self-perceptions regarding the ability to process music in an emotionally sophisticated manner are relevant to the model.

The finding that general emotional abilities such as EI, and more specific related skills such as recognition of facial expression (Petrides and Furnham, 2003), are involved in musical emotion recognition is perhaps of greater significance. While this is not a novel discovery, these results reinforce the idea that recognition of emotion within music is supported by general emotion processing mechanisms and appear consistent with a link between processes involved in recognition of emotions in speech and music (Juslin and Laukka, 2003) as put forward within the functionalist perspective of music and emotion (Juslin, 1997). The weak and non-significant relationship between MEDT test performance and vocal recognition ability shown in the current study is inconsistent with this explanation, however. This distinction could have resulted from a general discrepancy between the ease with which individuals are able to decode expressions in different modalities. Livingstone et al. (2015) have shown that vocal communication tends to be more difficult to decipher than facial expression. Similarly, Thompson (2010) reports that facial expression is generally more heavily relied on for emotional cues both within speech and music performance.

Divergence between test performance could also be a consequence of differences between the tasks used to measure musical and vocal recognition. The MEDT uses a 2-AFC task with two musical clips which can be played repeatedly, whereas the vocal recognition task from the ERI (Scherer and Scherer, 2011) makes use of a 5-AFC task where participants are required to select which of five emotions (the same four used in the MEDT plus “fearful”) they perceive a single non-verbal expression to convey, and are instructed to do so as quickly as possible. It is feasible that the requirement for participants to remember the vocal extract along with the added time pressure and the inclusion of fearful expressions in the vocal recognition task could have engaged memory mechanisms which may have contributed to the distinction between results. Correlations between recall of vocalizations and musical expressions are shown to be strongest when employing fearful stimuli, compared with happy and sad stimuli (Aubé et al., 2013), probably due to the greater evolutionary significance associated with the fear response. Thus, instead of reflecting a fundamental distinction between the mechanisms engaged in musical and vocal non-verbal processing, the current results are likely to be partially attributable to the disproportionate engagement of memory processes in the vocal recognition test when compared with the MEDT.

Perhaps equally puzzling is the equivocal influence of musical expertise; the present study considered alongside research conducted by Akkermans et al. (2018) contributes yet further ambiguity to the previous enquiries into musical and prosodic emotional expression recognition, which have so far proven inconclusive (Taruffi et al., 2017). While Akkermans et al. (2018) reported discovering a positive relationship between training and an emotional understanding of music performance, both experiments 2 and 3 of the current study revealed a negligible relationship. The current results could, however, be explained by the recruitment of samples that do not adequately represent the full range of musical training normally found in the general population (Müllensiefen et al., 2014). This is clear when considering the average MT score of participants in experiment 2 was 21.2 (SD = 10.7) and in experiment 3 was 18.6 (SD = 9) compared with the mean score of 26.5 (SD = 11.4) in the general population. Furthermore, the sample recruited for experiment 3 consisted exclusively of psychology undergraduate students who generally possess a restricted range of musical training background. As previously discussed, Lima and Castro (2011) argue that a limited level of musical expertise may lead to the lack of a measurable transfer effect on emotional ability. Investigation with a larger proportion of musically trained participants is therefore required to clarify the effect of musical training on the ability to discriminate emotions conveyed by music.

From a broader perspective, the successful operation of the test more generally, i.e., the fact that listeners were able to distinguish between basic emotions conveyed through music, supports the theoretical assumption that basic emotions can be portrayed through music performance (Juslin, 1995) as well as the applicability of discrete emotional constructs within the study of music and emotion, in spite of recent critique (Cespedes-Guevara and Eerola, 2018). However, it must still be considered that the stimuli used within the current experiment were specifically manipulated in order to portray these particular emotions, and this procedure is distinct from that which is likely to occur within a natural music performance. In a realistic setting, intrinsic structural aspects of the score would typically determine the intended emotional expression, and these emotive intentions would then be reflected by the musicians’ performance (Resnicow et al., 2004). The fact that only three performers were featured in the stimulus set used presents another limitation as performers may differ in terms of their technical skill (Gabrielsson and Juslin, 1996), as well as their interpretation of a given emotional expression (Akkermans et al., 2018). This is likely to impact upon the ease with which listeners are able to recognize intended expressions. Future studies should, therefore, aim toward including a wider range of stimuli that are more representative of music that one would typically encounter in everyday life, and feature a larger sample of performers.

## Conclusion

The potential for a musical performer to transmit emotional meaning and stimulate emotional-aesthetic experiences is a motivating factor behind listeners’ engagement with music. However, just as performers differ in expressive ability, it appears that some listeners may be better able to perceive performer-intended expression than others. This research contributes to an understanding of the origins of individual differences in music-perceived emotions, backing up previous findings that suggest that emotion-decoding ability is related to the ability to recognize facial expression of emotion and to self-reported emotional sensitivity to music. Furthermore, this study describes the development of a short and effective test of an individual’s capacity to identify emotions expressed through music performance. The test has acceptable psychometric qualities and is publicly available upon request.

## Data Availability

The datasets generated for this study are available on request to the corresponding author.

## Ethics Statement

This study was carried out in accordance with the recommendations of the ethics committee at the Department of Psychology, Goldsmiths, University of London, with online informed consent from all subjects. All subjects gave online consent in accordance with the BPS Code of Ethics and Conduct.

## Author Contributions

DM was responsible for conception of the test and study design, while CM was responsible for design, implementation, and data collection. Both authors contributed to the statistical analysis as well as to the production and editing of the manuscript.

## Conflict of Interest Statement

The authors declare that the research was conducted in the absence of any commercial or financial relationships that could be construed as a potential conflict of interest.

## Footnotes

1 The SoX manual can be found via the URL: http://sox.sourceforge.net/sox.html.
